# Novel rapid molecular diagnosis methods for comprehensive genetic analysis of 21-hydroxylase deficiency

**DOI:** 10.1186/s13023-024-03414-4

**Published:** 2024-10-28

**Authors:** Yanjie Xia, Feng Yu, Ying Bai, Lili Jiang, Panlai Shi, Zhengwen Jiang, Xiangdong Kong

**Affiliations:** 1 Genetics and Prenatal Diagnosis Center, Henan Engineering Research Center for Gene Editing of Human Genetic Disease, The First Affiliated Hospital of Zhengzhou University, Zhengzhou, China; 2 Genesky Diagnostics (Suzhou) Inc, Rm# 5F, Suite# C13, 218 Xinghu Street, SIP, Suzhou, Jiangsu China

**Keywords:** Congenital adrenal hyperplasia, 21-OHD, SNaPshot, CNVplex, Genetic analysis

## Abstract

**Background:**

Molecular analysis of the CYP21A2 gene is highly important for understanding the aetiology of 21-hydroxylase deficiency (21-OHD). The aim of this study was to use a novel approach named CNVplex, together with the SNaPshot assay and direct sequencing, to identify *CYP21A2* mutations efficiently and comprehensively. Targeted *CYP21A2* mutation analysis was performed in 113 patients and 226 parents. Large rearrangements of *CYP21A2* were characterized by CNVplex; twenty prevalent mutations, including nine common micro-conversions and eleven high-frequency mutations reported in the literature, were detected by SNaPshot; and rare mutations were investigated by direct sequencing.

**Results:**

Among the 113 21-OHD patients, 95.6% of the affected alleles were detected accurately by SNaPshot and CNVplex. Prevalent mutations were detected in 69.5% of the alleles; 62.4% of alleles contained pseudogene-derived micro-conversions, 1.8% contained nonpseudogene-derived mutations, and 5.3% contained complex variations resulting from multiple recombinations between *CYP21A2* and *CYP21A1P*. Large rearrangements were identified in 27.0% of the alleles, including five types (CH-1, CH-3, CH-4, CH-5 and CH-8) of chimeric *CYP21A1P/CYP21A2* genes. Two novel *CYP21A2* haplotypes and four *de novo CYP21A2* mutations were characterized. A rare haplotype with a c.955 C > T mutation in the duplicated *CYP21A2* gene was found in 0.9% of the probands and 33.3% of the parents. In addition, four parents were also diagnosed with 21-OHD.

**Conclusion:**

CNVplex and SNaPshot appear to be highly efficient and reliable techniques for use in a molecular diagnosis laboratory, and combined with direct sequencing based on locus-specific PCR, they might constitute a definitive way to detect almost all common and rare 21-OHD-related alleles.

**Supplementary Information:**

The online version contains supplementary material available at 10.1186/s13023-024-03414-4.

## Background

Congenital adrenal hyperplasia (CAH) due to 21-hydroxylase deficiency (21-OHD) is an autosomal recessive disorder characterized by impaired cortisol biosynthesis, with or without impaired aldosterone biosynthesis, accounting for ~ 95% of all CAH cases [1]. On the basis of the different clinical phenotypes resulting from the different mutations that affect 21-hydroxylase enzymatic activity, three subtypes of 21-OHD can be distinguished: salt-wasting CAH (SW-CAH), simple virilizing CAH (SV-CAH), and nonclassical CAH (NC-CAH). SW-CAH patients are characterized by a life-threatening salt-wasting adrenal crisis occurring within the first 3 weeks after birth if the disorder is not diagnosed and treated. In addition, affected female infants are generally identified by genital ambiguity. In SV-CAH patients, the residual activity of 21-hydroxylase (aldosterone is synthesized) results in external genitalia virilisation in females, and signs of precocious pseudopuberty develop before the 8th year in female and male patients [2]. Individuals with NC-CAH typically present in late childhood, adolescence, or early adulthood with signs and symptoms of excessive androgen secretion [3]. Throughout the lifespan, the primary goal of therapy for each patient is to restore the adrenal insufficiency of CAH to maintain normal plasma volume and physiological balance. The measurement of 17-hydroxyprogesterone (17-OHP) with or without adreno-cortico-tropic-hormone (ACTH) stimulation is used to establish a preliminary diagnosis of CAH due to 21-OHD. Molecular genetic testing for *CYP21A2* gene mutations is another important method for 21-OHD diagnosis.

Steroid 21-hydroxylase is a cytochrome P450 enzyme that uses P450 oxidoreductase to transport electrons from Nicotinamide adenine dinucleotide phosphate (NADPH) and catalyses the hydroxylation of the adrenal steroid hormones progesterone and 17-hydroxyprogesterone at the C21 position of steroid biomolecules [4]. The human steroid 21-hydroxylase consists of 495 amino acids and has a molecular weight of 52 kDa; the protein localizes to the endoplasmic reticulum and is exclusively expressed on the adrenal gland [5]. The whole region of the human *CYP21A2* gene (OMIM*613815) spans 3.35 kb and is located on chromosome 6p21.3. In this region, four tandemly arranged genes—serine/threonine kinase (*RP*), complement (*C4*), steroid 21-hydroxylase (*CYP21*), and tenascin (*TNX*)—are organized as a genetic unit known as the RCCX module (*RP*-*C4*-*CYP21*-*TNX*) [6]. The RCCX module is characterized by high homology between the functional genes (*RP1*, *CYP21A2*, and *TNXB*) and their corresponding pseudogenes (*RP2*, *CYP21A1P* and *TNXA*). Copy number variations for this RCCX locus have been previously described; bimodular variation is the standard, whereas monomodular, trimodular and quadrimodular variations are rare cases [7–9]. Owing to the high homology and tandem repeats of the RCCX module, misalignment may occur during meiosis, leading to large rearrangements including large deletions, duplications, and the formation of nonfunctional chimeric genes.

Cases of 21-OHD with a genetic cause arise from mutations in the *CYP21A2* gene, which shares 98% exon homology and 96% intron homology with the nonfunctional pseudogene *CYP21A1P* [10]. Deleterious defects harboured in the pseudogene can be transferred to the functional gene, producing common mutations that account for approximately 95% of all *CYP21A2* mutations observed in 21-OHD patients [11]. Among these common mutations, 75% are micro-conversions, and 20–25% are gene deletions or conversions that generate chimeric *CYP21A1P/CYP21A2* genes via unequal meiotic crossover [12, 13]. Other rare unequal meiotic crossover arrangements can generate duplicate *CYP21A2* genes, which have been found in Swedish [14, 15], Spanish [8], Tunisian [16] and other populations [17, 18], and are associated mainly with the presence of severe c.955 C > T or c.293–13 A/C > G mutations in one of the *CYP21A2* genes. The highly polymorphic complexity of this locus, the high homology of the pseudogene and the presence of *CYP21A2* gene duplications complicate the determination of disease and carrier status, making precise genotyping of 21-OHD challenging.

To date, a variety of molecular techniques have been used for genetic analysis of 21-OHD. Gene deletions and large conversions are traditionally detected by Southern hybridization, but this method has been replaced by multiplex ligation-dependent probe amplification (MLPA) [19], a technique widely described in recent years. Strategies for point variant detection include methods such as allele-specific PCR, single-stranded conformation polymorphisms, liquid chromatography, SNaPshot assay and minisequencing; some of these methods have been reviewed by Pignatelli D et al. [20]. Detection methods such as allele-specific PCR or MLPA can identify both the *CYP21A2* gene downstream of the *TNXA* gene and the *CYP21A2* gene next to the *TNXB* gene, leading to misinterpretation of the genotyping results. However, a locus-specific PCR approach based on four sets of primers could not only accurately and faultlessly detect these two *CYP21A2* genes but also determine whether genomic rearrangement or deletion has occurred [21, 22].

In this paper, we present a new approach named CNVplex, which is based on MLPA and can analyse up to 160 gene loci in a single array. In addition, twenty prevalent mutations in *CYP21A2* were analysed by SNaPshot to obtain simple and fast results. CNVplex together with SNaPshot can determine the phase of deletions/duplications/micro-conversions and further distinguish attenuated chimaeras from class chimaeras. The aim of our study was to perform a comprehensive *CYP21A2* mutation analysis in 113 21-OHD patients and their parents using CNVplex, SNaPshot assay and Sanger sequencing based on locus-specific PCR in a sequential strategy to provide fast, reliable and comprehensive genetic diagnoses.

## Results

### Mutational spectrum in 21-OHD patients

The summaries of genotypes and clinical phenotypes are shown in additional file 1, and the mutational frequencies are shown in Table 1. In 113 patients, 55 genotypes were found, which were multifarious. Prevalent mutations was detected in 69.5% (157/226) of all alleles; 62.4% (141/226) of alleles contained pseudogene-derived micro-conversions, 1.8% (4/226) contained non pseudogene-derived mutations, and 5.3% (12/226) contained complex variations resulting from multiple recombinations between *CYP21A2* and *CYP21A1P*. Large rearrangements were found in 27.0% (61/226) of the alleles, including 8.0% (18/226) with large deletions, 17.7% (40/226) with *CYP21A1P*/*CYP21A2* chimaeras, and 1.3% (3/226) with large-scale conversions. Five of the nine known *CYP21A1P*/*CYP21A2* chimaeras were identified, including CH-1, CH-3, CH-4, CH-5 and CH-8. In conclusion, when CNVplex was combined with the SNaPshot assay, 95.6% (216/226) of affected alleles in 21-OHD patients could be detected, and no false-positive results were obtained, indicating 100% sensitivity and specificity compared with MLPA together with Sanger sequencing. The remaining 4.4% of the alleles (10/226) were not identified by the SNaPshot or CNVplex assays, and direct sequencing of the *CYP21A2* gene was conducted. In these 10 unidentified alleles, rare point mutations were detected, including seven missense mutations (c.1054G > A, c.1063 C > T, c.874G > A, c.1273G > A, c.1379 C > A c.1081 C > T and c.1423 C > T) and one splice site mutation (c.651 + 2T > G).

**Table 1:** Mutation frequency in 113 patients with 21-hydroxylas deficiency

Detection methods	Type of variation	DNA sequence	Protein effect	Alleles (*n* = 226)	Frequency (%)
SNaPshot	Micro-conversions	c.293–13 A/C > G	^a^NA	69	30.5
		c.518T > A	p.I173N	28	12.4
		c.1069 C > T	p.R357W	12	5.3
		c.955 C > T	p.Q319X	9	4.0
		c.1451_1452delGGinsC	p.R484Pfs*58	7	3.1
		c.844G > T	p.V282L	5	2.2
		c.92 C > T	p.P31L	4	1.8
		E6cluster (c.710T > A; c.713T > A; c.719T > A)	p.I236N; p.V237E; p.M239K	5	2.2
		c.332_339delGAGACTAC	p.G111Vfs*21	2	0.9
	Non pseudogene -derived mutations	c.274 A > G	p.R92G	2	0.9
		c.292 + 1G > A	^a^NA	1	0.4
		c.1450dupC	p.R484P	1	0.4
	Complex variations	c.293–13 A/C > G + c.332_339delGAGACTAC	^a^NA	2	0.9
		c.293–13 A/C > G + c.1451_1452delGGinsC	^a^NA	1	0.4
		c.518T > A + c.710T > A + c.713T > A + c.719T > A	^a^NA	1	0.4
		c.710T > A + c.713T > A + c.719T > A + 923dupT + 955 C > T	^a^NA	2	0.9
		c.710T > A + c.713T > A + c.719T > A + 844G > T + 923dupT	^a^NA	1	0.4
		c.923dupT + c.955 C > T + 1069 C > T	^a^NA	2	0.9
		c.923dupT + c. 955 C > T	^a^NA	1	0.4
		c.293–13 A/C > G + c.332_339delGAGACTAC + c.518T > A + c.710T > A + c.713T > A + c.719T > A + c.844G > T + c.923dupT + c.955 C > T + c1451_1452delGGinsC	^a^NA	1	0.4
		c.518T > A + c.710T > A + c.713T > A + c.719T > A + c.844G > T + c.923dupT + c.955 C > T + c.1451_1452delGGinsC	^a^NA	1	0.4
CNVplex	Large scale deletions	Deletion	^a^NA	18	8.0
		CH-1	^a^NA	17	7.5
		CH-8	^a^NA	11	4.9
		CH-3	^a^NA	8	3.5
		CH-4	^a^NA	3	1.3
		CH-5	^a^NA	1	0.4
	Large scale conversions	*CYP21A2* downstream the TNXA pseudogene	^a^NA	3	1.3
Sanger sequencing	Rare point mutations	c.1054G > A	p.E352K	3	1.3
		c.1063 C > T	p.R355C	1	0.4
		c.874G > A	p.G292S	1	0.4
		c.1273G > A	p.G425S	1	0.4
		c.1379 C > A	p.P460H	1	0.4
		c.1081 C > T	p.P361S	1	0.4
		c.651 + 2 T > G	^a^NA	1	0.4
		c.1423 C > T	p.Q475X	1	0.4

^a^NA, not applicable

### **De novo** mutations

In general, mutant alleles in 21-OHD patients are inherited from their carrier biological parents. The mutated alleles of the 21-OHD patients in 113 families were traced back to their parents in 96.5% (109/113) of families, whereas *de novo* mutations were found in four families (Table 2). The patients in F10 and F102 inherited the c.1069 C > T or c.844G > T mutation, respectively, from their fathers. Because the c.293–13 A/C > G mutation in these two patients was not found in their respective mothers, this *CYP21A2* aberration was classified as a *de novo* mutation. The proband in F84 had a *de novo* c.874G > A mutation and a c.293–13 A/C > G mutation inherited from one of her parents. Patients in F113 carried both a *de novo* deletion and a *de novo* c.293–13 A/C > G mutation. The father of F113 had a monomodular chromosome with a deletion of *CYP21A1P*, whereas the mother did not exhibit any mutation (Additional file 2).

**Table 2:** Four patients with *de novo* mutations

Fmilly Number	Gender	Phenotype	proband genotype	Maternal genotype	Paternal genotype	De novo mutaions
F10	Female	SV	c.1069 C > T; c.293–13 A/C > G	N	c.1069 C > T	c.293–13 A/C > G
F84	Female	SW	c.293–13 A/C > G; c.874G > A	c.293–13 A/C > G	c.293–13 A/C > G	c.874G > A
F102	Female	NC	c.293–13 A/C > G; c.844G > T	N	c.844G > T	c.293–13 A/C > G
F113	Male	SW	Deletion; c.293–13 A/C > G	N	N	Deletion and c.293–13 A/C > G

SV: simple virilizing; SW: salt-wasting; NC: non-classical; N, normal

### Two novel haplotypes

**Fig. 1:**
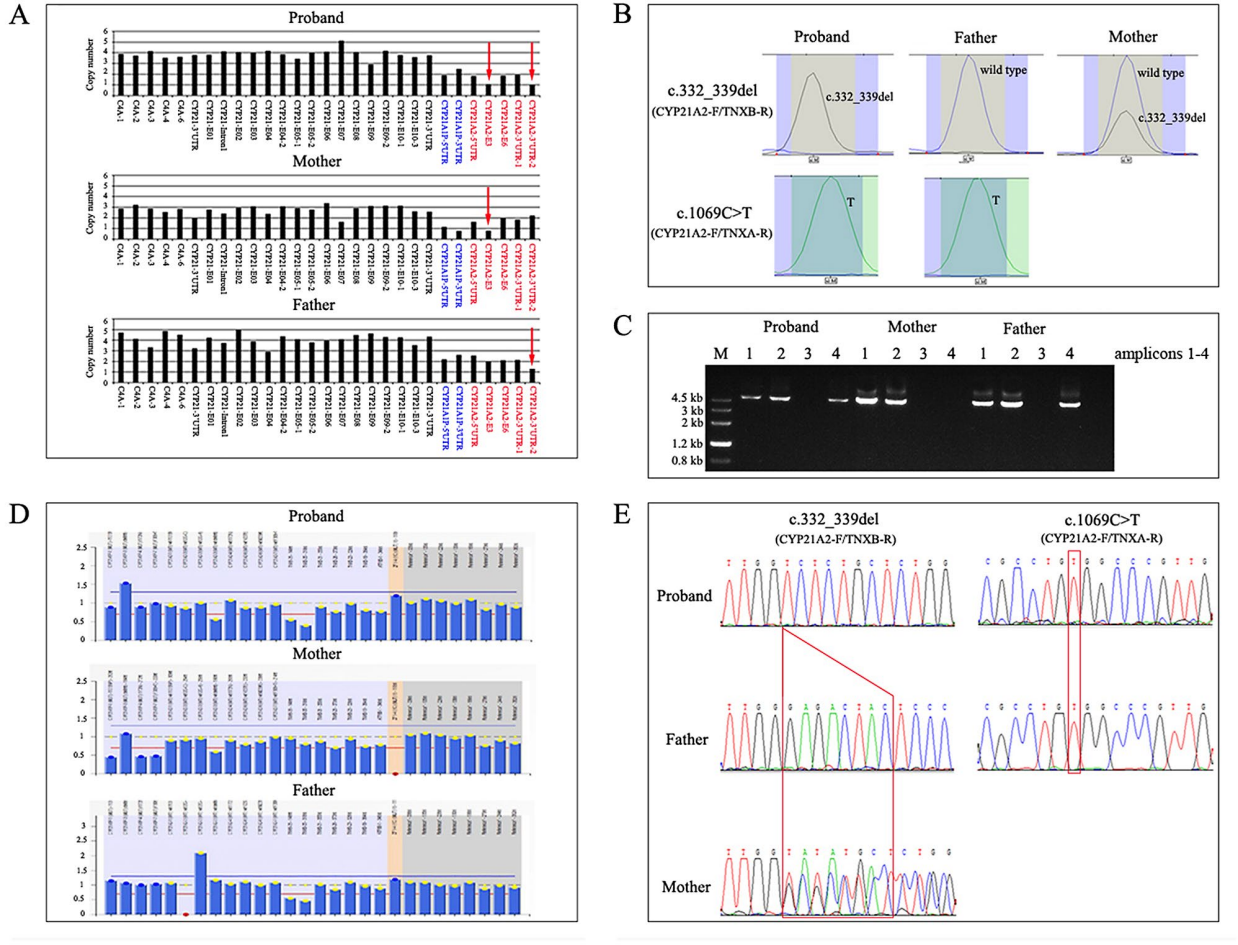
Molecular analysis of *CYP21A2* in F80. **(A)** CNVplex of *CYP21A2* locus for the proband and parents. **(B)** SNaPshot assay of *CYP21A2* locus for the proband and the parents. **(C)** locus-specific PCR amplicons of the proband and parents. **(D)** MLPA analysis of *CYP21A2* locus for the proband and parents. (E) Sanger sequencing of the *CYP21A2* gene for the proband and parents

The proband in F80 was diagnosed with SW-CAH based on clinical phenotype. CNVplex showed a reduced copy number in the E3 and 3’-UTR regions of the patient but a reduced copy number in the E3 region of her mother and in the 3’-UTR of her father (Fig. 1A). SNaPshot assays of *CYP21A2* revealed that this patient was homozygous for c.332_339delGAGACTAC, whereas her mother was a heterozygote and her father was normal (Fig. 1B). Locus-specific PCR amplification revealed that this patient and her father were positive for amplicon 1, 2 and 4, and her mother was positive for amplicon 1 and 2 (Fig. 1C). Further analysis of amplicon 4 via the SNaPshot assay revealed that the patient and her father were both homozygous for c.1069 C > T (Fig. 1B). The MLPA and Sanger sequencing results in this family were consistent with those of the CNVplex and SNaPshot assays (Fig. 1D and E). In conclusion, CNVplex combined with SNaPshot analysis revealed that this patient inherited the allele with the c.332_339delGAGACTAC mutation from her mother and the allele with a c.1069 C > T mutation in the *CYP21A2* gene downstream of the *TNXA* gene from her father.

**Fig. 2:**
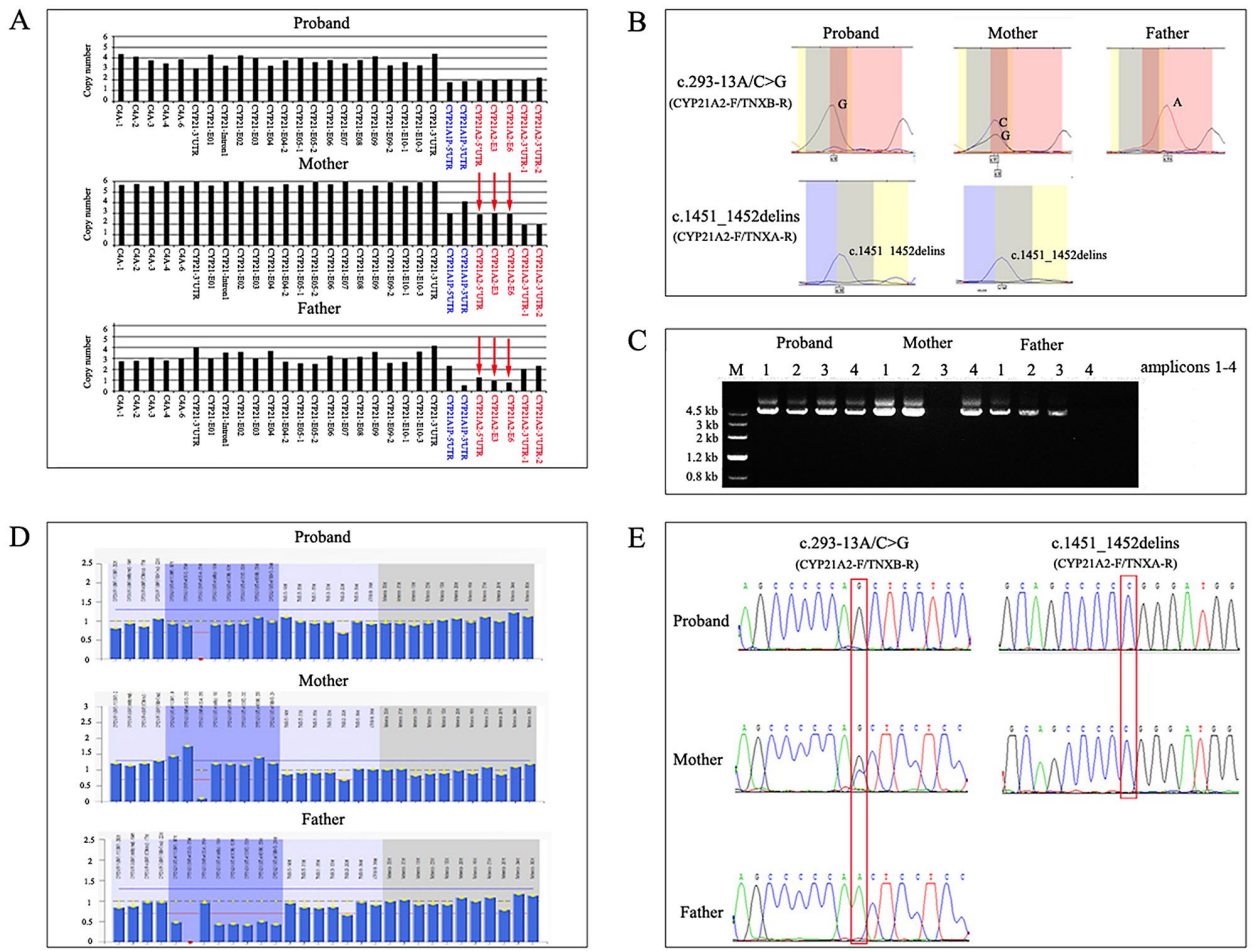
Molecular analysis of *CYP21A2* in F88. **(A)** CNVplex of *CYP21A2* locus for the proband and parents. **(B)** SNaPshot assay of *CYP21A2* locus for the proband and the parents. **(C)** locus-specific PCR amplicons of the proband and parents. **(D)** MLPA analysis of *CYP21A2* locus for the proband and parents. **(E)** Sanger sequencing of the *CYP21A2* gene for the proband and parents

The proband in F88 was diagnosed with SV-CAH based on clinical phenotype. The results of the first genetic investigation at another hospital revealed a genotype‒phenotype discrepancy, indicating that the patient was a heterozygous c.293-13A/C > G mutation carrier according to MLPA and targeted sequencing of the *CYP21A2* gene. Because her mother was pregnant again, a second genetic evaluation was requested. Therefore, both parents were enrolled in the study. CNVplex analysis of her mother revealed three copies of the 5’-UTR, E3 and E6 and two copies of the 3’-UTR detected with the *CYP21A2*-specific probes, indicating the existence of an additional *CYP21A2* gene. CNVplex of her father revealed one copy of the 5’-UTR, E3 and E6 and two copies in the 3’-UTR detected with the *CYP21A2*-specific probes, indicating a 30-Kb deletion of the *CYP21A2* gene. However, CNVplex revealed two copies of the *CYP21A2* gene in this patient, suggesting that the false-negative results were caused by duplication-masked deletion (Fig. 2A). The SNaPshot assay revealed a homozygous c.293–13 A/C > G mutation in the patient, a heterozygous mutation in her mother and a wild-type allele in her father (Fig. 2B). Locus-specific PCR amplification revealed that this patient was positive for amplicons 1–4, while her mother was positive for amplicons 1, 2, and 4, and her father was positive for amplicons 1, 2, and 3 (Fig. 2C). Further analysis of amplicon 3 revealed a CH-8 chimaera in the patient and her father, and analysis of amplicon 4 revealed a mutated (c.1451_1452delGGinsC) *CYP21A2* gene downstream of the *TNXA* gene in the patient and her mother (Fig. 2B). The MLPA and Sanger sequencing results in this family were consistent with those of the CNVplex and SNaPshot assays (Fig. 2D and E). Finally, we identified this patient as a compound heterozygote with CH-8 in one allele and a mutated (c.293–13 A/C > G) *CYP21A2* gene next to the *TNXB* gene and a mutated (c.1451_1452delGGinsC) *CYP21A2* gene downstream of the *TNXA* gene in the other allele.

### Haplotypes with the c.955 C > T mutation and duplicated *CYP21A2* gene

Rare haplotypes with c.955 C > T mutations and a duplicated *CYP21A2* gene have been reported in different populations [16, 23]; however, this haplotype remains unknown in the Chinese population. Here, we present an analysis of the c.955 C > T mutation in Chinese 21-OHD patients and healthy carriers. In this study, a total of nine patients and twelve healthy carriers with the c.955 C > T mutation were identified. Further analysis of the *CYP21A2* gene dosage by CNVplex and MLPA revealed that one patient (1/9, 11.1%) and four carriers (4/12, 33.3%) harboured three copies of *CYP21A2*, suggesting a duplication event (Table 3).

**Table 3:** Patients and carriers with c.955 C > T mutation

MLPA and CNVplex			
Group	Number of duplicated CYP21A2	Number of normal CYP21A2	Percent
patients	1	8	11.1%
carriers	4	8	33.3%

### Four parents were incidentally diagnosed with 21-OHD

Among the 226 parents studied, four were also identified as 21-OHD patients according to the separation of Mendelian laws. Two pathogenic mutations were found in the fathers of F6, F111 and F112 and the mother of F110, from whom the probands inherited one of the mutations, suggesting that the two mutations in these parents were located on separate alleles, which was confirmed by genetic analysis of the grandparents (Table 4). Interestingly, all four patients were clinically asymptomatic, were born after marriage through natural means of conception and were identified as having 21-OHD in the course of family analysis of a 21-OHD index patient.

**Table 4:** Four parents were diagnosed for 21-OHD patients

Fmilly Number	Proband genotype	Maternal genotype	Paternal genotype	Genotype of grandmother	Genotype of grandfather
F6	CH-8; c.92 C > T	CH-8 and duplicated *CYP21A2*	CH-2; c.92 C > T	c.92 C > T	CH-2
F110	c.274 A > G; c.293–13 A/C > G	c.293–13 A/C > G; c.844G > T	c.274 A > G	c.293–13 A/C > G	c.844G > T
F111	CH-4; c.293–13 A/C > G	c.293–13 A/C > G	CH-4; c.844G > T	CH-4	c.844G > T
F112	CH-1; c.293–13 A/C > G	CH-1	c.293–13 A/C > G; c.518T > A	c.293–13 A/C > G	c.518T > A

## Discussion

Molecular analysis of the *CYP21A2* gene is highly important for understanding the aetiology of 21-OHD in both clinical diagnosis and basic science. However, the comprehensive molecular diagnosis of 21-OHD is often complex and laborious, requiring exhaustive work. In this study, CNVplex and SNaPshot were used for high-throughput, sensitivity and specificity analyses with small amounts of DNA samples and nonradioactive probes, allowing the simultaneous study of different samples in the same experiment. Overall, 95.6% of affected alleles in 21-OHD patients could be detected accurately using SNaPshot and CNVplex. Indeed, more than 90% of the disease-causing alleles carried common mutations derived from *CYP21A1P* through intergenic recombination [24, 25]. In addition, five (CH-1, CH-3, CH-5, CH-8) *CYP21A1P/CYP21A2* chimaeras were easily distinguished, and a mutant *CYP21A2* gene downstream of the *TNXA* gene was also identified through SNaPshot analysis of amplicon 4. Third, we identified two novel *CYP21A2* haplotypes and four *de novo CYP21A2* mutations. Intriguingly, four parents were also found to have 21-OHD. Our data suggest that exhaustive genetic testing may need to be performed in 21-OHD patients to provide accurate genetic counselling.

Most *CYP21A2*-deficient alleles carry preexisting mutations in the homologue pseudogene, which are usually inherited from carrier parents. In this study, among 226 alleles detected in patients, four *de novo CYP21A2* aberrations were identified, representing 2.2% of the informative alleles. This percentage was consistent with previous reports suggesting that *de novo* germline mutations in *CYP21A2* occur in 1–2% of CAH alleles [11]. The presence of parental haplotypes with variable lengths of the RCCX module might predispose offspring to *de novo* mutations in *CYP21A2*. Unequal meiotic crossing between bimodular and monomodular [26] and between bimodular and trimodular RCCX units [27] has been suggested as a possible cause of *de novo* mutations in offspring. Given that the father in F113 had a deletion of *CYP21A1P*, we propose that an unequal crossover generated *a de novo* deletion identified in the proband of F113. Compared with *de novo* mutations of large deletions or conversion, *de novo* point mutations are rare. Previously, a *de novo* p.I172N mutation was reported by Finkielstain et al. [28]. In this study, we identified a *de novo* point mutation in three 21-OHD patients with bimodular RCCX units in parental haplotypes. Both p.I172N and c.293–13 A/C > G are common micro-conversion positions at which *de novo* mutations might be prone to occur.

An unequal meiotic crossover event usually produces trimodular haplotypes of the RCCX module with duplication of the *CYP21A2* gene, carrying one copy of the *CYP21A1P* pseudogene and two copies of the *CYP21A2* gene [29]. In most cases, the *CYP21A2* gene downstream of the *TNXA* gene exhibited a wild-type sequence or the c.293–13 A/C > G mutation, whereas the *CYP21A2* gene next to *TNXB* contained the c.955 C > T mutation [8, 16, 23]. The trimodular haplotype explained the genotype‒phenotype discrepancy in individuals of different families, suggesting the importance of trimodular haplotype assessment in CAH genetic diagnosis [30, 31]. In this study, we used the specific paired primers CYP21A2-F/TNXA-R or CYP21A2-F/TNXB-R to amplify the *CYP21A2* gene downstream of *TNXA* or next to *TNXB*, respectively, followed by the SNaPshot assay to examine the mutational locus. In our group, 2.6% (3/113) of 21-OHD patients carried a *CYP21A2* gene downstream of *TNXA*, and three haplotypes were identified, including a well-known haplotype and two novel haplotypes that have never been described. Given these novel findings, it is essential to consider the presence of these two unusual haplotypes as a rare genetic condition in the Chinese population. A similar event should be carefully considered when a genotype/phonotype discrepancy is revealed during the molecular diagnosis of 21-hydroxylase deficiency.

The trimodular haplotype harboring a duplicated *CYP21A2* gene with a c.955 C > T aberration has been widely reported, with a high frequency in certain ethnic groups [16]. In the present study, we found that 33.3% of the heterozygous carriers had this specific haplotype. Discrimination between a normal and a CAH allele is important. If two *CYP21A2* genes are present, the allele bearing the c.955 C > T mutation represents a CAH allele. If three *CYP21A2* genes are present, family analysis must be performed to determine the localization of the c.955 C > T mutation. Individuals carrying a c.955 C > T mutation on the allele with two functional *CYP21A2* genes are not CAH carriers but rather harbor a functional normal allele. This information is crucial in genetic counselling as well as in prenatal diagnosis. To avoid false-positive genotyping, it is highly important to be aware of this unique haplotype.

Interestingly, four parents were also identified to have 21-OHD. All of them were asymptomatic before participating in this study and never received relevant therapy; presumably, all of them might have had NC-CAH. NC-CAH is a relatively common disorder regardless of ethnicity, but most cases are never diagnosed, especially in males, and most are identified during genetic screening [32, 33]. The NC-CAH phenotype is conferred by mild mutations in the *CYP21A2* gene, resulting in a 30–50% reduction in the activity of the enzyme [33]. The fathers in F6 and F111 and the mother in F110 were compound heterozygous; all parents carried either the c.92 C > T or c.844G > T mutation, causing a mild enzyme deficiency associated with NC-CAH. The father in F112 was compound heterozygous for c.293–13 A/C > G and c.518T > A, which is expected to be SV-CAH based on the genotype-phenotype described previously by Wedell et al. [34] and Speiser et al. [35], however, a small portion of patients present the NC phenotype [36]. Similar to our research, which involved comprehensive genetic testing of 145 unrelated patients with CAH, 10/249 (4%) parents were diagnosed with NC-CAH. Ten parents were compound heterozygotes for a severe mutation and a mild mutation. However, their affected children inherited the severe mutation [37]. Previous studies have shown that the risk of an NC-CAH patient having a child with classic CAH is 1 to 2% [38, 39]; thus, it is essential to genotype the partners of patients with a severe mutation to predict the risk of classical CAH offspring and offer preconception genetic counselling.

## Conclusion

CNVplex is sensitive and specific in its ability to detect large rearrangements of the *CYP21A2* gene when combined with the SNaPshot assay and direct sequencing based on locus-specific PCR, allowing us to determine all 21-OHD genotypes quickly, accurately and reliably. A limitation of this study is that the *CYP21A1P/CYP21A2* chimaera gene can be distinguished by the presence of common mutations, but the accurate location of recombination breakpoints in the rearrangement products needs to be identified by sequencing.

## Methods

### Study subjects

We studied 113 unrelated patients with 21-OHD, representing 226 unrelated affected alleles; we also studied all the parents to assess genetic segregation. Phenotypic forms of 21-OHD were determined based on clinical manifestation and hormonal evaluations as previously described [40]. A commercial genomic DNA extraction kit in Tubes (Qiagen, Germany) was used to isolate DNA from the blood. Quantified by spectrophotometer at 260 nm and stored at -20 ℃ until use. This study was approved by the Ethics Committee of the First Affiliated Hospital of Zhengzhou University (NO. KS-2018-KY-36). Written informed consent for genetic testing was given by adult subjects and parents of participating adolescents.

### CNVplex analysis

In this study, large rearrangements of *CYP21A2* were measured by CNVplex assay, a high-throughput multiplex copy number variant analysis method recently developed by Genesky Biotechnologies. This kit contains 41 different probes with amplification products between 97 and 192 bp. Five probes are specific for the *CYP21A2* gene and recognize the 5’UTR region, E3, E6 and 3’UTR region, respectively (Additional file 3). Furthermore, two specific probes are located in the 5’UTR and 3’UTR region of *CYP21A1P*, 17 probes for homologous sequences of *CYP21A1P* and *CYP21A2* gene and 5 probes for homologous sequences of *C4A* and *C4B* gene. Finally, 12 probes specific for human genes are included as controls for copy number quantification. The schematic of the experimental principle and workflow of this technique has been illustrated [41].

In CNVplex, peak ratios between 0.8–1.2, 1.7–2.3, 2.7–3.3 and 3.6–4.4 are considered as high-quality data of one copy, two copies, three copies and four copies of target gene, respectively. Signals of the probes detecting the *CYP21A2* wildtype sequence should be in the main focus, while other probes are only included for accurate result interpretation. Based on the copy number measurements for all target sequences, large rearrangements of *CYP21A2* can be estimated. DNA samples from standard healthy individuals have two copies in all five *CYP21A2*-specific probes. Other situations are as follows: 1) increased copy number in all *CYP21A2*-specific probes means *CYP21A2* duplication; 2) decreased copy number detected in all *CYP21A2*-specific probes means *CYP21A2* deletion; 3) increased copy number in 5’ region while normal copy number in 3’ region of *CYP21A2*-specific probes means existence of another *CYP21A2* gene downstream the *TNXA* gene; 4) decreased copy number in 5’ region while normal copy number in 3’ region of *CYP21A2*-specific probes means the existence of *CYP21A1P/CYP21A2* chimeric genes.

### SNaPshot assay

Point mutations were analysed by SNaPshot assay (Genesky Biotechnologies Inc.). Primers were designed on Primer3 software v.0.4.0 and specific sequences were analysed using BLAST software (US National Library of Medicine) (Additional file 4). Twenty prevalent mutations were selected, including nine pseudogene-derived micro-conversions (c.92 C > T, c.293 A/C > G, c.332_339delGAGACTAC, c.518T > A, E6 cluster, c.844G > T, c.923dupT, c.955 C > T, c.1069 C > T) and other eleven high-frequency pathogenic mutations (c.274 A > G, c.292 + 1G > A, c.449G > C, c.949 C > T, c.1225 C > T, c.1226G > T, c.1279 C > T, c.1450dupC, c.1451G > C, c.1451_1452delGGinsC, c.1455delG) reported in the literature. The workflow of this technique was described previously [42].

### Locus-specific PCR amplification and Sanger sequencing

**Fig. 3:**
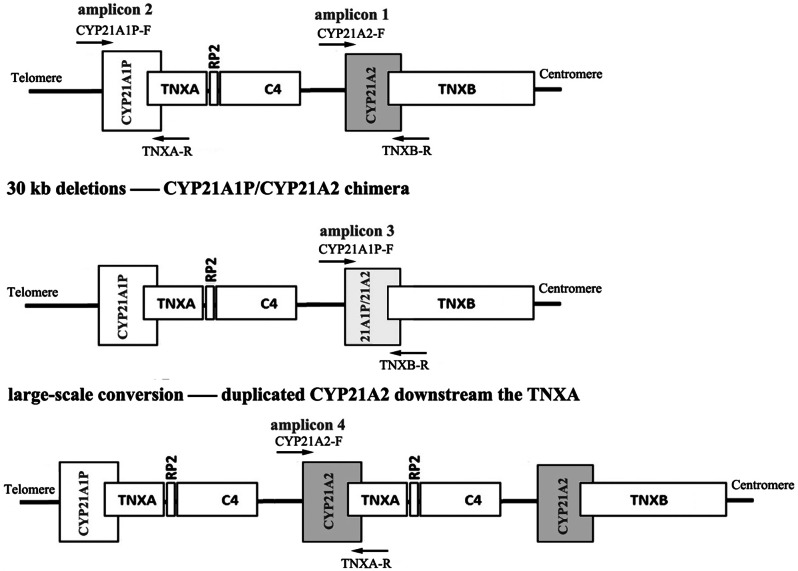
Strategy for locus-specific PCR amplication to detect *CYP21A1P*, *CYP21A2*, the common 30-kb deletion and large-scale conversion alleles

PCR amplifications were performed with four sets of locus-specific primers to amplify the *CYP21A2* next to *TNXB* (primers: CYP21A2-F/TNXB-R), the *CYP21A1P* (primers: CYP21A1P-F/TNXA-R), the 30-kb deletions generating *CYP21A1P/CYP21A2* chimaera (primers: CYP21A1P-F/TNXB-R), and large-scale conversion generating *CYP21A2* downstream the *TNXA* (primers: CYP21A2-F/TNXA-R) (Fig. 3). PCR amplifications of CYP21A2-F/TNXB-R contained genome sequence hg38 chr6:32038297–32,043,834, and amplifications of CYP21A1P-F/TNXA-R contained genome sequence hg38 chr6: 32,005,565–32,011,003. The expected sizes of the PCR products are as follows: *CYP21A2*-5.5 kb, *CYP21A1P*-5.4 kb, *CYP21A1P*/*CYP21A2* chimaera-5.5 kb and large-scale conversion-5.4 kb. PCR reactions were performed in 20 µL total volume containing 1x HotStarTaq buffer, 3.0 mM Mg^2+^, 0.3 mM dNTP, 1 U HotStarTaq polymerase (Qiagen Inc.), 10 ng DNA and 0.2 µM of each primer. PCR following touch-down cycling program: 95 ℃ for 2 min; 11 cycles of 94 ℃ for 20 s, 65 ℃ for 30 s (reduced 0.5 ℃ each cycle), and 72 ℃ for 90s followed by 24 cycles of 94 ℃ for 20 s, 59 ℃ for 30 s, and 72 ℃ for 2 min with a final 72 ℃ extension for 10 min and a hold at 4 ℃.

### Mutation analysis of each *CYP21A2* genes

Frequent mutations in *CYP21A2* gene downstream of *TNXB* or next to *TNXA* were further analyzed by SNaPshot assay with amplicon 1 or 4, respectively. Rare mutations in *CYP21A2* gene were detected by direct sequencing with internal primers on an ABI 3730XL genetic analyzer (Applied Biosystems). All primers used for direct sequencing are shown in Additional file 5. Besides, the results of SNaPshot assay were also evaluated by direct sequencing. Rare mutations identified by Sanger sequencing were selected for data interpretation with a cut-off value of < 0.01 in 1000 Genomes Project, HapMap and dbSNP as reported [43–46]. Classification of these mutations has been done according to the variant interpretation guidelines of American College of Medical Genetics and Genomics (ACMG).

### Classification of *CYP21A1P/CYP21A2* chimeric genes

**Fig. 4:**
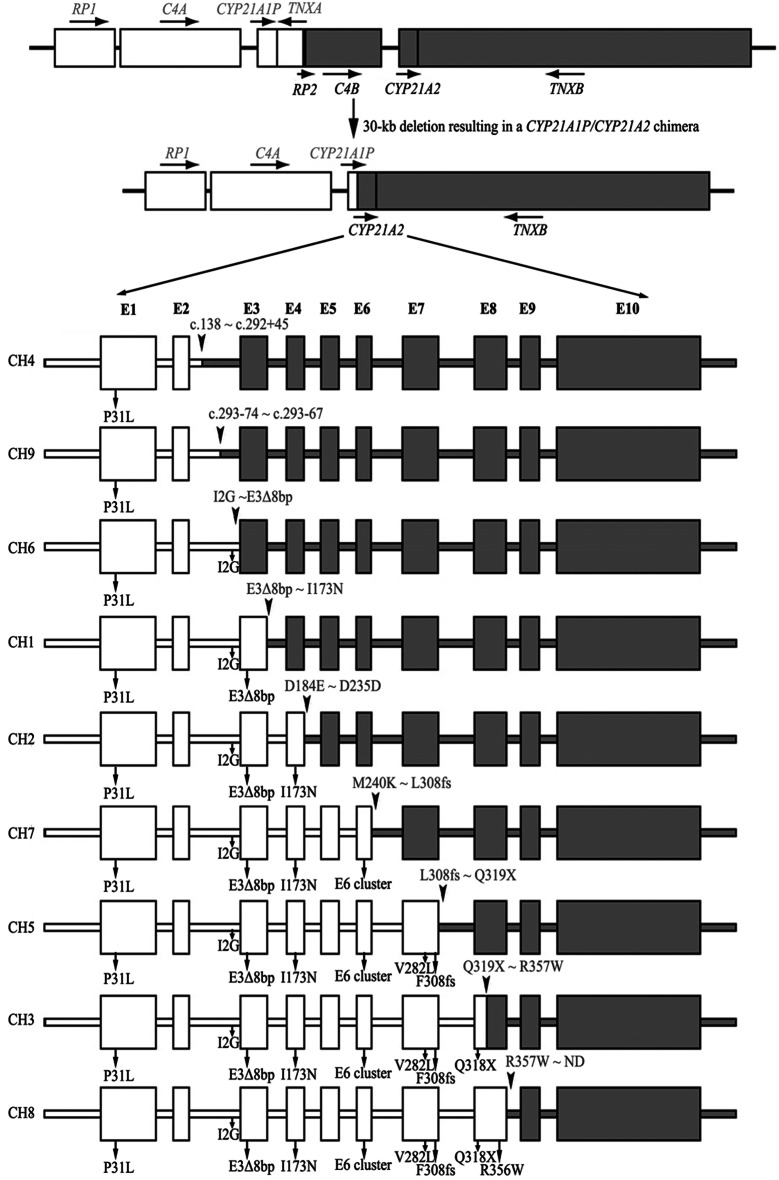
Carriership of common micro-conversions in nine types of chimaera (CH1-CH9), the common micro-conversions were indicated by arrows, and the junction site were indicated by arrowheads. I2G: c.293–13 A/C > G, ND: downstream site was not determined owing to a lack of distinguishable variants between *CYP21A1P* and *CYP21A2*

*CYP21A1P*/*CYP21A2* chimeric genes were further analyzed by SNaPshot assay with amplicon 3 to identify the chimeric type. Classic chimaeras can be easily distinguished by the carriership of common micro-conversions [47] (Fig. 4). Attenuated chimaeras, known as CH-4 and CH-9, both could be detected the only c.92 C > T mutation by SNaPshot assay. Sequence analysis of amplicon 3 with the primer A04R (Additional file 5) was performed to determine the breakpoints, which located between c.138 and c.292 + 45 in CH-4 while between c.293 − 74 and c.293 − 67 in CH-9.

### MLPA

Copy number and gene conversion analyses was performed by MLPA with the SALSA MLPA Kit P050-B2 (MRC Holland) as previously described [40] to evaluate the performance of CNVplex.

### Kinship testing

Once *de novo* mutations were found, kinship testing was performed between the probands and their parents. The Goldeneye™ 20 A STR kit was used to perform multiplex STR amplification on SimpliAmp Thermal Cycler (Thermo Fisher Scientific, Waltham, MA, USA). Electrophoretic separation of amplification products was run on an ABI-3130XL Genetic Analyzer, and analyzed by GeneMarker software for automated profiling. Paternity index (PI) values were calculated according to the technical specification for the paternity test (GB/T 37223 − 2018).

## Electronic supplementary material

Below is the link to the electronic supplementary material.

**Supplementary Material 1:** Genotype and phenotype in 113 Chinese 21-OHD patients. SW: salt-wasting forms, SV: simple virilizing forms, NC: non-classical forms, ND: undefined of clinical forms.

**Supplementary Material 2:** Probe information used for CNVplex^®^

**Supplementary Material 3:** Primer sequences used for SNaPshot assay

**Supplementary Material 4:** MLPA analysis of *CYP21A2* locus for the proband and parents in F113

**Supplementary Material 5:** The sequences of primers for direct sequencing

## Data Availability

All data generated or analysed during this study are included in this published article [and its supplementary information files].

## Abbreviations

CAH: Congenital Adrenal Hyperplasia
21-OHD: 21-Hydroxylase deficiency
SBA: Southern blot analysis
ASO-PCR: Allele-specific PCR
SSCP: Single-stranded conformation polymorphisms
