# Supplementary figures and images for: Novel rapid molecular diagnosis methods for comprehensive genetic analysis of 21-hydroxylase deficiency
Source: Orphanet J Rare Dis. 2024 Oct 28;19:397. doi: 10.1186/s13023-024-03414-4 (PMC11514819; doi:10.1186/s13023-024-03414-4)

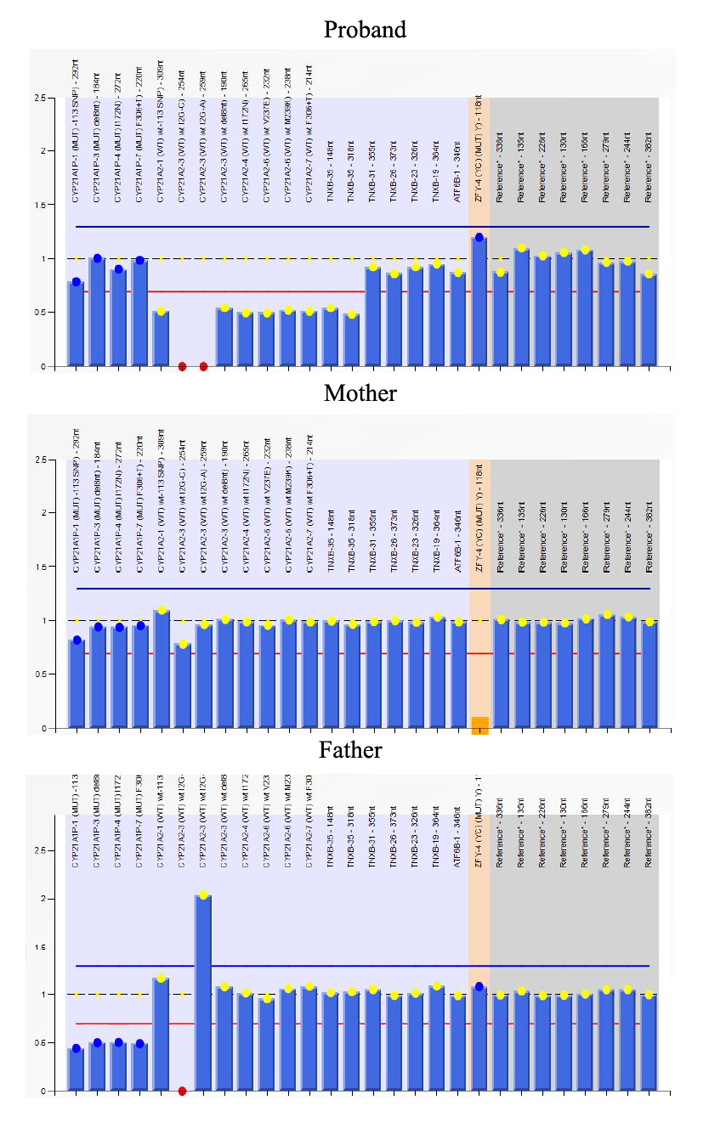

Supplement: Supplementary file 1 — Supplementary Material 1: Genotype and phenotype in 113 Chinese 21-OHD patients. SW: salt-wasting forms, SV: simple virilizing forms, NC: non-classical forms, ND: undefined of clinical forms. [file 13023_2024_3414_MOESM1_ESM.tif]

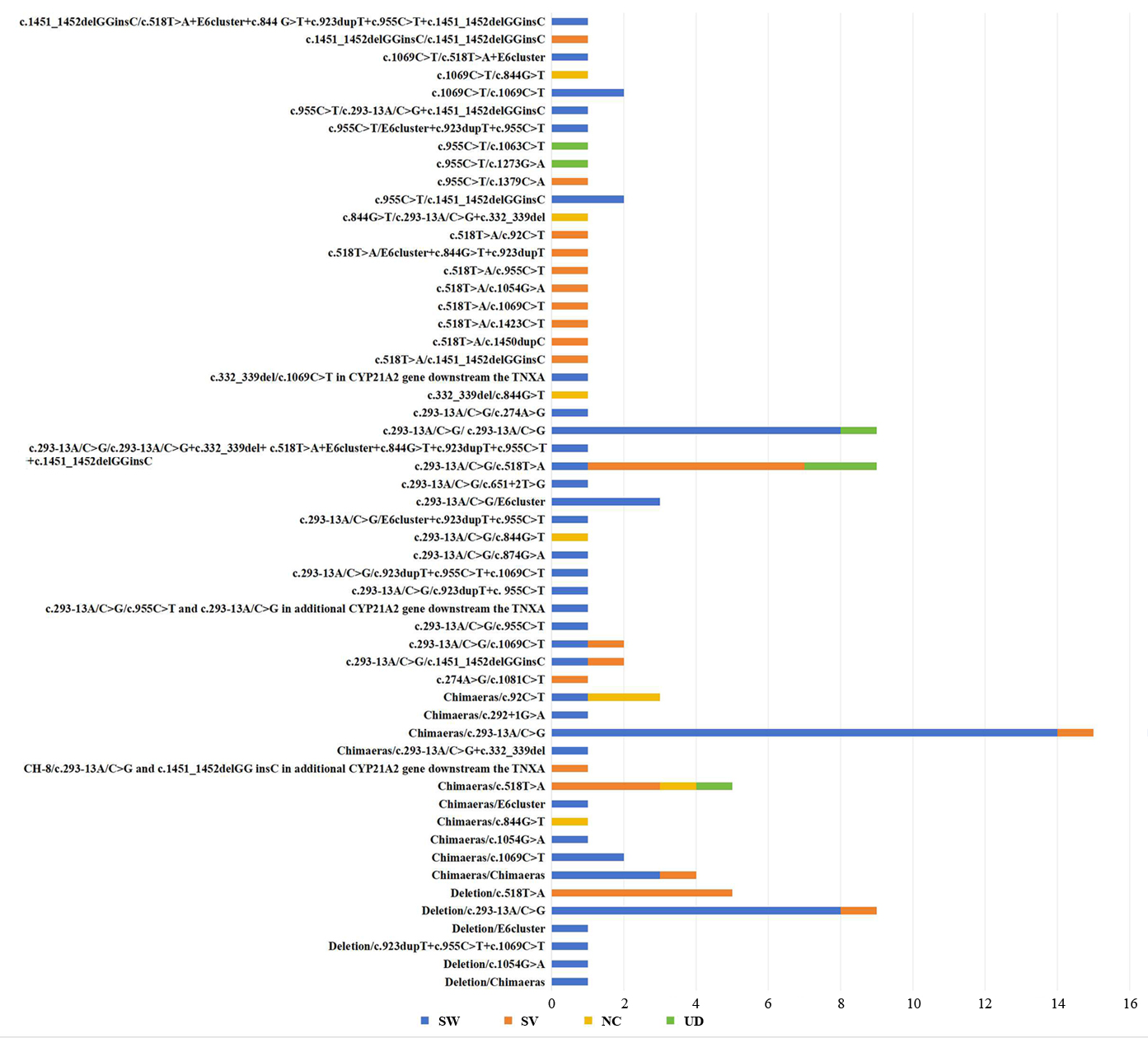

Supplement: Supplementary file 4 — Supplementary Material 4: MLPA analysis of CYP21A2 locus for the proband and parents in F113 [file 13023_2024_3414_MOESM4_ESM.jpg]
