# Supplementary material for: Novel rapid molecular diagnosis methods for comprehensive genetic analysis of 21-hydroxylase deficiency
Source: Orphanet J Rare Dis. 2024 Oct 28;19:397. doi: 10.1186/s13023-024-03414-4 (PMC11514819; doi:10.1186/s13023-024-03414-4)
Supplement: Supplementary file 2 — Supplementary Material 2: Probe information used for CNVplex® [file 13023_2024_3414_MOESM2_ESM.docx]

**Additional file 3.** Probe information used for CNVplex^®^

| Item | Name | Label | Group | Probe name of 5' end | Probe length of 5' end | Probe name of 3' end | Probe length of 3' end | Fragment length (nt) | Probe binding region | Chromosomal position of probe binding region (GRCh37) |
| --- | --- | --- | --- | --- | --- | --- | --- | --- | --- | --- |
| Control probes | R20FAR1REF02 | FAM | S1 | R20FAR1REF02_5F3 | 47 | R20FAR1REF02_3F2 | 42 | 97 | GCCACTGGGGCTTTAGGAATGGTACTTCGCAGCTCAGTGCAGCC | Chr3:87027376-87027419 |
|  | R20FAR1REF06 | FAM | S1 | R20FAR1REF06_5F2 | 50 | R20FAR1REF06_3F2 | 47 | 105 | GTGCCGGAGAAGATGATTCATGACTCTAGCCCTGCTTTTTCTCTCCCG | Chr9:75301852-75301899 |
|  | R20FAR1REF10 | FAM | S1 | R20FAR1REF10_5F2 | 57 | R20FAR1REF10_3F2 | 58 | 123 | TTCATCTGGATCCATGACGATGGACAAGTAGCCTGTCTTCAGTTCCCCTC | Chr4:55964383-55964432 |
|  | R20FAR1REF15 | FAM | S1 | R20FAR1REF15_5F2 | 68 | R20FAR1REF15_3F2 | 64 | 140 | GGGATGTGGTGTACACCTACCATCAGTTTATAGAGGCTGCTAAATCGACCTGCG | Chr9:37746603-37746656 |
|  | R20FAR2REF18 | FAM | L1 | R20FAR2REF18_5F3 | 47 | R20FAR2REF18_3F2 | 44 | 142 | TGCCTAGCTGTGTGACATGATTGGCATCCCCAGTGTGGGACCATG | Chr6:51589994-51590038 |
|  | R20FAR2REF22 | FAM | L1 | R20FAR2REF22_5F2 | 51 | R20FAR2REF22_3F2 | 50 | 152 | TGAGTTCACATCACAGCGAGGGATTCAGGGTATTGAATCTTGTGGGGG | Chr3:97554704-97554751 |
|  | R20FAR2REF26 | FAM | L1 | R20FAR2REF26_5F2 | 58 | R20FAR2REF26_3F2 | 62 | 171 | GGCTTTCTTCCCATCCCAGTCAGCCTGAAAAATTGTCTCTATCAGGGAAA | Chr2:84500198-84500247 |
|  | R20FAR2REF30 | FAM | L1 | R20FAR2REF30_5F3 | 69 | R20FAR2REF30_3F2 | 68 | 188 | AAGGCCAGGCCTGTATTTAGTTAGTGGTAATGATCTCGTTAGGGGTGTGAATGG | Chr20:20868408-20868461 |
|  | R20NAR2REF24 | FAM | S1 | R20NAR2REF24_5F2 | 54 | R20NAR2REF24_3F2 | 53 | 115 | CTGGAGTGAGGGGAAGAAGCTGTTACAGAAGTGGAATGGTTTCTGGTGG | Chr12:27952126-27952174 |
|  | R20PAR1REF13 | FAM | L1 | R20PAR1REF13_5F2 | 64 | R20PAR1REF13_3F2 | 65 | 180 | TCACACAGAGTCACCCTCCTTCTGAAGTGCTTTGCTTATAGCAGCCTGAACC | Chr17:26232654-26232705 |
|  | R20VAR2REF23 | FAM | S1 | R20VAR2REF23_5F2 | 62 | R20VAR2REF23_3F2 | 63 | 133 | GGCTGGGAACCTCAAGAGTGAGGCATCCTTGAGCAACTCTGGGTTCTACA | Chr10:46009655-46009704 |
|  | R20VAR2REF27 | FAM | L1 | R20VAR2REF27_5F2 | 55 | R20VAR2REF27_3F2 | 55 | 161 | CCTTTGACTGGTTTGTGGCCATCTGTTGTCCTCTCTGTTACGGCCTCATC | Chr11:59516471-59516520 |
| Probes for homologous sequences | C4A-1 | FAM | L1 | C4A-1_5R2 | 53 | C4A-1_3R2 | 53 | 157 | TGGAGGTCAGTAATGCCCATATTCAGCTTCTCCAGGGCGTCCTGGAACT | Chr6:31952141-31952093 Chr6:31984879-31984831 |
|  | C4A-2 | FAM | L1 | C4A-2_5F3 | 65 | C4A-2_3F2 | 65 | 181 | GTCTTCTCCTGGGTCTGTTCCTGAAGTCCAGGACATTCAGCAAAACACAG | Chr6:31959249-31959298 Chr6:31991987-31992036 |
|  | C4A-3 | FAM | S1 | C4A-3_5F | 55 | C4A-3_3F | 55 | 118 | GCAGATCGTGTTCATGAATCGAGAGCCCAAGAGGACCCTGACCTC | Chr6:31959838-31959882 Chr6:31992576-31992620 |
|  | C4A-4 | FAM | L1 | C4A-4_5F | 49 | C4A-4_3F | 50 | 150 | GTGGTGGCTCGAGGGTCCTTCGAATTCCCTGTGGGAGATGC | Chr6:31962419-31962459 Chr6:31995157-31995197 |
|  | C4A-6 | FAM | L1 | C4A-6_5R2 | 57 | C4A-6_3R2 | 58 | 166 | GTGTTCATGAAAGCGGACACAGCAGTGCTTCCAGCTTCATGGTTCC | Chr6:31970403-31970358 Chr6:32003140-32003095 |
|  | CYP21-3’UTR | FAM | L1 | CYP21AP01_5F2 | 69 | CYP21AP01_3F | 67 | 187 | TGATGTGGAACCAGAAAGCTGACTCTGGATGCAGGAAAAAGGTCAGG | Chr6:31973184-31973230 Chr6:32005919-32005965 |
|  | CYP21-E01 | FAM | L1 | CYP21A2-E01_5R2 | 48 | CYP21A2-E01_3R2 | 48 | 147 | AGGGCCCTGAGGTGCCACTTATAGCTCAAGAGCCCCAGCCATCCCT | Chr6:31973450-31973405 Chr6:32006184-32006139 |
|  | CYP21-Intron1 | FAM | S1 | CYP21A2-E01-E02_5F | 56 | CYP21A2-E01-E02_3F | 56 | 120 | GGTCCTCTCTCCGCTGACGCTGCTTTGGCTGTCTCCCAGATGT | Chr6:31973726-31973768 Chr6:32006460-32006502 |
|  | CYP21-E02 | FAM | L1 | CYP21A2-E02_5F2 | 52 | CYP21A2-E02_3F2 | 51 | 154 | GATGTGGTGGTGCTGAACTCCAAGAGGACCATTGAGGAAGCCATGGT | Chr6:31973764-31973810 Chr6:32006498-32006544 |
|  | CYP21-E03 | FAM | L1 | CYP21A2-E03_5F | 58 | CYP21A2-E03_3F | 59 | 168 | AGTGGTGGAGCAGCTGACCCAGGAGTTCTGTGAGGTAAGGCTGGGC | Chr6:31974256-31974301 Chr6:32006992-32007037 |
|  | CYP21-E04 | FAM | S1 | CYP21A2-E04_5F | 61 | CYP21A2-E04_3F | 61 | 130 | CCTGTGGCCATTGAGGAGGAATTCTCTCTCCTCACCTGCAGCATCA | Chr6:31974421-31974466 Chr6:32007157-32007202 |
|  | CYP21-E04-2 | FAM | L1 | CYP21A2-E04-2_5R2 | 62 | CYP21A2-E04-2_3R | 64 | 177 | AGGGGCTGTGAGGCACCTTGATCTTGTCTCCGAAGGTGAGGTAACAG | Chr6:31974514-31974468 Chr6:32007250-32007204 |
|  | CYP21-E05-1 | FAM | L1 | CYP21A2E05-1_5F2 | 65 | CYP21A2E05-1_3F2 | 57 | 173 | GTATCCAGGAGGTGTTAAAAACCTGGAGCCACTGGTCCATCCAAATTGTG | Chr6:31974618-31974667 Chr6:32007354-32007403 |
|  | CYP21-E05-2 | FAM | S1 | CYP21A2E05-2_5F2 | 60 | CYP21A2E05-2_3F2 | 60 | 128 | TGATTCCCTTTCTCAGGGTGAGGACCTGGAGCCTAGACACCCCTG | Chr6:31974672-31974716 Chr6:32007408-32007452 |
|  | CYP21-E06 | FAM | S1 | CYP21A2-E06_5F2 | 50 | CYP21A2-E06_3F2 | 50 | 108 | AGGGAGAGGCTCCTTCCCACAGCTGCATTCTCATGCTTCCTGC | Chr6:31974742-31974784 Chr6:32007478-32007520 |
|  | CYP21-E07 | FAM | S1 | CYP21A2-E07_5F2 | 58 | CYP21A2-E07_3F2 | 59 | 125 | CAGGCCAGTGGAGGGACATGATGGACTACATGCTCCAAGGGGTG | Chr6:31975059-31975102 Chr6:32007795-32007838 |
|  | CYP21-E08 | FAM | S1 | CYP21A2-E08_5F3 | 52 | CYP21A2-E08_3F2 | 51 | 111 | CCTACAAGGACCGTGCACGGCTGCCCTTGCTCAATGCCACCAT | Chr6:31975515-31975557 Chr6:32008250-32008292 |
|  | CYP21-E09 | FAM | L1 | CYP21A2-E09_5F | 62 | CYP21A2-E09_3F | 62 | 175 | CTACGACATCCCTGAGGGCACAGTCATCATTCCGAACCTCCAAGGC | Chr6:31975719-31975764 Chr6:32008454-32008499 |
|  | CYP21-E09-2 | FAM | S1 | CYP21A2-E09-2_5F2 | 65 | CYP21A2-E09-2_3F2 | 62 | 135 | GAGACGGTCTGGGAGAGGCCACATGAGTTCTGGCCTGGTATGTGG | Chr6:31975777-31975821 Chr6:32008512-32008556 |
|  | CYP21-E10-1 | FAM | S1 | CYP21A2-E10-1_5F2 | 53 | CYP21A2-E10-1_3F2 | 52 | 113 | CTTCGTGGTGCTGACCCGACTGCTGCAGGCCTTCACGCTGCTG | Chr6:31976005-31976047 Chr6:32008740-32008782 |
|  | CYP21-E10-3 | FAM | S1 | CYP21A2-E10-3_5F | 65 | CYP21A2-E10-3_3F | 65 | 138 | GAGCGAAAGTTTCTTGGTCTCAGCTTCATTTCCGTGAAGGGCACCGAGAA | Chr6:31976410-31976459 Chr6:32009145-32009194 |
|  | CYP21-3’UTR | FAM | L1 | CYP21ATE500_5F7 | 63 | CYP21ATE500_3F6 | 66 | 132 | GAGATGTTCCCAAAACCATGGGCATAGTCCTCCCAGTCCCTCCA | Chr6:31977325-31977368 Chr6:32010060-32010103 |
| Probes for *CYP21A1P* | CYP21A1P-5'UTR | FAM | S1 | CYP21A1PUS2C-5F | 45 | CYP21A2US2D-3F | 45 | 98 | CGGTGGGAAGGCACCTGAGGGTGGGGTCAAGGGAGGC | Chr6:31973345-31973381 |
|  | CYP21A1P-3'UTR | FAM | L1 | CYP21A1PDSX_5F2 | 73 | CYP21A1PDSX_3F2 | 72 | 192 | TTTTCTTGgCTCCCACCTGGAGTTTCTGGGTCCGGGCCCG | Chr6:31978747-31978786 |
| Probes for *CYP21A2* | CYP21A2-5’UTR | FAM | S1 | CYP21A2US2D-5F | 47 | CYP21A2US2D-3F | 45 | 100 | TCGGTGGGAGGGTACCTGAAGGTGGGGTCAAGGGAGGC | Chr6:32006078-32006115 |
|  | CYP21A2-E3 | FAM | L1 | CYP21A2E03-1\|5F | 54 | CYP21A2E03-1\|3F | 54 | 159 | GACCTGTCCTTGGGAGACTACTCCCTGCTCTGGAAAGCCCACAA | Chr6:32006897-32006940 |
|  | CYP21A2-E6 | FAM | L1 | CYP21A2E06-1\|5F3 | 57 | CYP21A2E06-1\|3F3 | 56 | 164 | CCATAGAGAAGAGGGATCACATCGTGGAGATGCAGCTGAGGCAGC | Chr6:32007563-32007607 |
|  | CYP21A2-3’UTR-1 | FAM | S1 | CYP21A2DS3C-5R2 | 48 | CYP21A2DS3C-3R2 | 47 | 103 | cacaggcttcctcaccacgggtgagatggactgggacccg | Chr6:32011545-32011506 |
|  | CYP21A2-3’UTR-2 | FAM | L1 | CYP21A2DS2-5F2 | 47 | CYP21A2DS2-3F2 | 47 | 145 | CTCACTCTCCTCAAAGCCTCGGACCGAGACCACGGTCACCTCATA | Chr6:32011551-32011595 |
