# Supplementary material for: Novel rapid molecular diagnosis methods for comprehensive genetic analysis of 21-hydroxylase deficiency
Source: Orphanet J Rare Dis. 2024 Oct 28;19:397. doi: 10.1186/s13023-024-03414-4 (PMC11514819; doi:10.1186/s13023-024-03414-4)
Supplement: Supplementary file 3 — Supplementary Material 3: Primer sequences used for SNaPshot assay [file 13023_2024_3414_MOESM3_ESM.docx]

**Additional file 4.** Primer sequences used for SNaPshot assay

| Primer name | Mutation | Exon | Sequence (5’-3’ direction) | Primer length (base) | AddNts | Wild type | Nucleotide and size of peak | | | | | |
| --- | --- | --- | --- | --- | --- | --- | --- | --- | --- | --- | --- | --- |
| CYP21A2:c.274A>GSF | c.274A>G | E02 | TAGTGGGCAGACTTTGCTGGC | 21 | GA | A | G | 33.5 | A | 34.6 |  |  |
| CYP21A2:c.955C>TSR | c.955C>T | E08 | TTTTTAGTTCGTGGTCTAGCTCCTCCT | 27 | GA | G | G | 36 | A | 37.94 |  |  |
| CYP21A2:c.1069C>TSR | c.1069C>T | E08 | TTTTTTTTTTTTTTTTTTTGCTAAGGGCACAACGGGCC | 38 | GA | G | G | 42.06 | A | 43.3 |  |  |
| CYP21A2:c.1226G>TSR | c.1226G>T | E10 | TTTTTTTTTTTTTTTTTTTTTTTTTTTTTTTTTTTTCTTGCCTGGCTCCAGGAAG | 55 | CA | C | C | 60.9 | A | 61.5 |  |  |
| CYP21A2:c.92C>TSR2 | c.92C>T | E01 | TTTTTTTTTTTTTTTTTTTTTTTTTTTTTTTTTTTTTTTTTTTTTTTTGAAGCCCGGGGCAAGAGGC | 67 | GA | G | G | 70.9 | A | 92.8 |  |  |
| CYP21A2:c.449G>CSF | c.449G>C | E04 | TTTTTTTTTTTTTTTTTTTCTGAACTCACACTGTTTCTCCACAGC | 45 | GC | G | G | 49.4 | C | 50.5 |  |  |
| CYP21A2:c.1450dupCSF | c.1450dupC | E10 | TTTTTTTTTTTTTTTTTTTTTTTTTTTTTTTTTTTTAAGTGCGGCTGCAGCCCC | 54 | GC | G | G | 56.7 | C | 57.5 |  |  |
| CYP21A2:c.293-13C>GSR | c.293-13A/C>G | Intron 2 | TTTTTTTTTTTTTTTTTTTTTTTTTTTTTTTTTTTTACCAGCTTGTCTGYAGGAGGAG | 58 | GTC | T | G | 62.23 | C | 63.45 | T | 62.5 |
| CYP21A2:c.1455delGSR | c.1455delG | E10 | TTTTTTTTTTTTTTTTTTTTTTTTTTTTTTTTTTTTTTTTTTTCTGTGGGCCCCCATCCCC | 62 | GC | C | G | 65.84 | C | 66.5 |  |  |
| CYP21A2:c.1452G>CSR | c.1452G>C | E10 | TTTTTTTTTTTTTTTTTTTTTTTTTTTTTTTTTTTTTTTTTTTTTTTGCTGTGGGCCCCCATCCC | 65 | GC | C | G | 69.3 | C | 70 |  |  |
| CYP21A2:c.332_339delSR1 | c.332_339delGAGACTAC | E03 | TTTTTTTTTTTTTTTTTTTTTTTTTTTTTTTTTTTTTTTTTTTTTTTTTTTTGTGGGCTTTCCAGAGCAGRGA | 73 | GC | G | G | 78.5 | C | 78.8 |  |  |
| CYP21A2:c.332_339delSF3 | c.332_339del_NEW | E03 | TTTTTTTTTTTTTTTTTTTTTTTTTTTTTTTTTTTTTTTTTTTTTTTTTAACTACCCGGACCTGTCCTTGG | 71 | GT | G | G | 74.84 | T | 77 |  |  |
| CYP21A2:c.949C>TSF | c.949C>T | E08 | TCATTCCCCAGATTCAGCAG | 20 | CT | C | C | 33.9 | T | 36 |  |  |
| CYP21A2:c.1225C>TSF | c.1225C>T | E10 | TTTTTTTTTTTTGTCCACCCGCCCGCAGAT | 30 | CT | C | C | 38 | T | 41 |  |  |
| CYP21A2:c.1279C>TSF | c.1279C>T | E10 | TTTTTTTTTTTTTTTTTGGCCTTCGGCTGCGGTGCC | 36 | CT | C | C | 42.3 | T | 45.5 |  |  |
| CYP21A2:c.518T>ASF | c.518T>A | E04 | TTTTTTTTTTTTTTTTTTTTTCTCCTCACCTGCAGCATCA | 40 | AT | T | A | 46.6 | T | 47.8 |  |  |
| CYP21A2:c.710T>ASR | c.710T>A | E06 | TTTTTTTTTTTTTTTTTTTGCTGCCTCAGCTGCWTCTCCWCG | 42 | AT | A | A | 49 | T | 50.6 |  |  |
| CYP21A2:c.713T>ASR | c.713T>A | E06 | TTTTTTTTTTTTTTTTTTTTTTTTTTCTGCCTCAGCTGCWTCTCC | 45 | AT | A | A | 51.24 | T | 52.74 |  |  |
| CYP21A2:c.719T>ASR | c.719T>A | E06 | TTTTTTTTTTTTTTTTTTTTTTTTTTTTTCTTGTGCTGCCTCAGCTGC | 48 | AT | A | A | 53.6 | T | 55 |  |  |
| CYP21A2:c.923dupTSF | c.923dupT | E07 | TTTTTTTTTTTTTTTTTTTTTTTTTTTTTTTTCTCCTGGGCCGTGGTTTTTTT | 53 | GT | G | G | 59.14 | T | 61 |  |  |
| CYP21A2:c.844G>TSF3 | c.844G>T | E07 | tttttttttttttttttttttttttttttttttttTGGACAGCTCCTGGAAGGGCAC | 57 | G/T | G | G | 60.55 | T | 62.5 |  |  |
| CYP21A2:c.332_339delSF | c.332_339del | E03 | TTTTTTTTTTTTTTTTTTTTTTTTTTTTTTTTTTTTTTTTTTTTTCCGGACCTGTCGTTGGTC | 63 | T | - | - | - | T | 70 |  |  |
| CYP21A2:c.292+1G>ASR | c.292+1G>A | I02 | TTTTTTTTTTTTTTTTTTTTTTTTTTTTTTTTTTTTTTTTTTTTTTTTTTAAAAATGCCCCCRGCCCTTA | 70 | CT | C | C | 74.5 | T | 75.8 |  |  |
