# Supplementary material for: Novel rapid molecular diagnosis methods for comprehensive genetic analysis of 21-hydroxylase deficiency
Source: Orphanet J Rare Dis. 2024 Oct 28;19:397. doi: 10.1186/s13023-024-03414-4 (PMC11514819; doi:10.1186/s13023-024-03414-4)
Supplement: Supplementary file 5 — Supplementary Material 5: The sequences of primers for direct sequencing [file 13023_2024_3414_MOESM5_ESM.docx]

**Additional file 5.** The sequences of primers for direct sequencing

| **Primers** | **Sequence (5’-3’)** | **Location** |
| --- | --- | --- |
| A01F | CGGGTCGGTGGGAGGGTACCTGAA | 5’-UTR region |
| A02F | CCAAGAGGACCATTGAGGAAG | Exon 2 |
| A03R | GGTGGGAGGATCATTTGAGA | Intron 2 |
| A04R | GAATTCCTCCTCAATGGCCAC | Exon 4 |
| A05F | TGCCTGCCTATTACAAATGTATCC | Exon 5 |
| A06R | CATGAGAATGCAGCTGTGGGAAG | Intron 5 |
| A07F | GGTTGGGGATGAGTGAGGAAAGC | Exon 8 |
| A08R | CTTGGAGGTTCGGAATGATGACTGT | Exon 9 |
